# Supplementary material for: Inhibitors of Lipoxygenase and Cyclooxygenase-2 Attenuate Trimethyltin-Induced Neurotoxicity through Regulating Oxidative Stress and Pro-Inflammatory Cytokines in Human Neuroblastoma SH-SY5Y Cells
Source: Brain Sci. 2021 Aug 24;11(9):1116. doi: 10.3390/brainsci11091116 (PMC8468241; doi:10.3390/brainsci11091116)
Supplement: Supplementary file 1 [file brainsci-11-01116-s001.zip › brainsci-1326867-supplementary.pdf]

Supplemental Figure S1

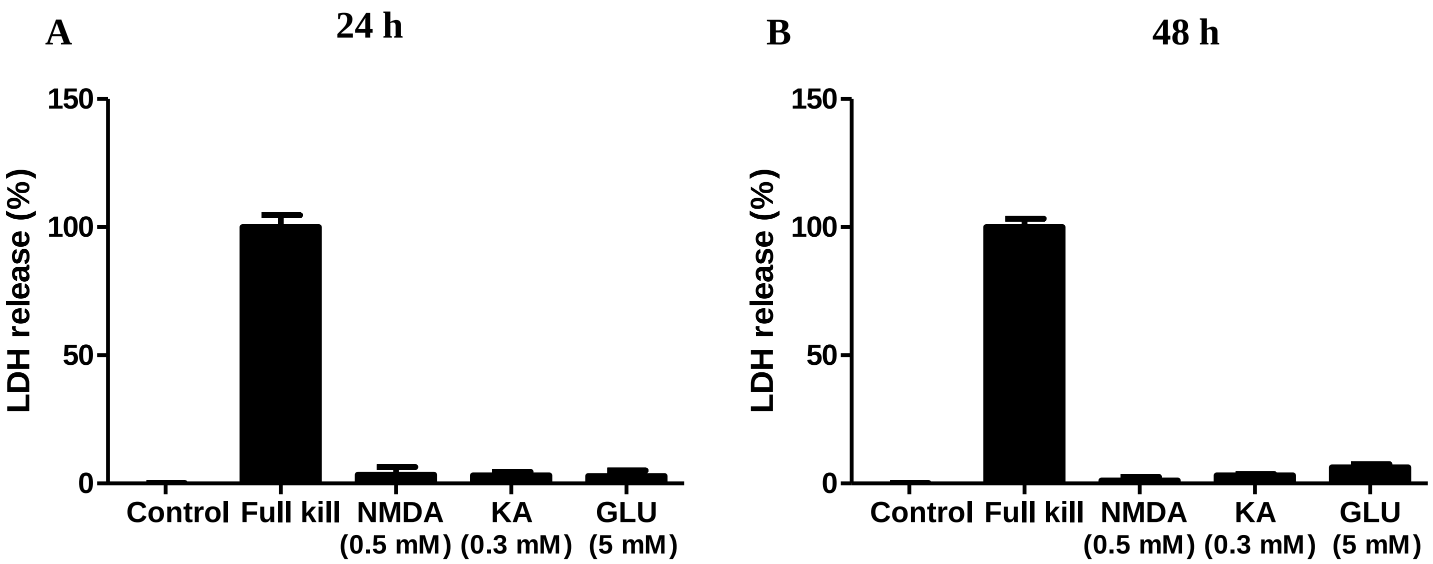

Supplemental Figure S2

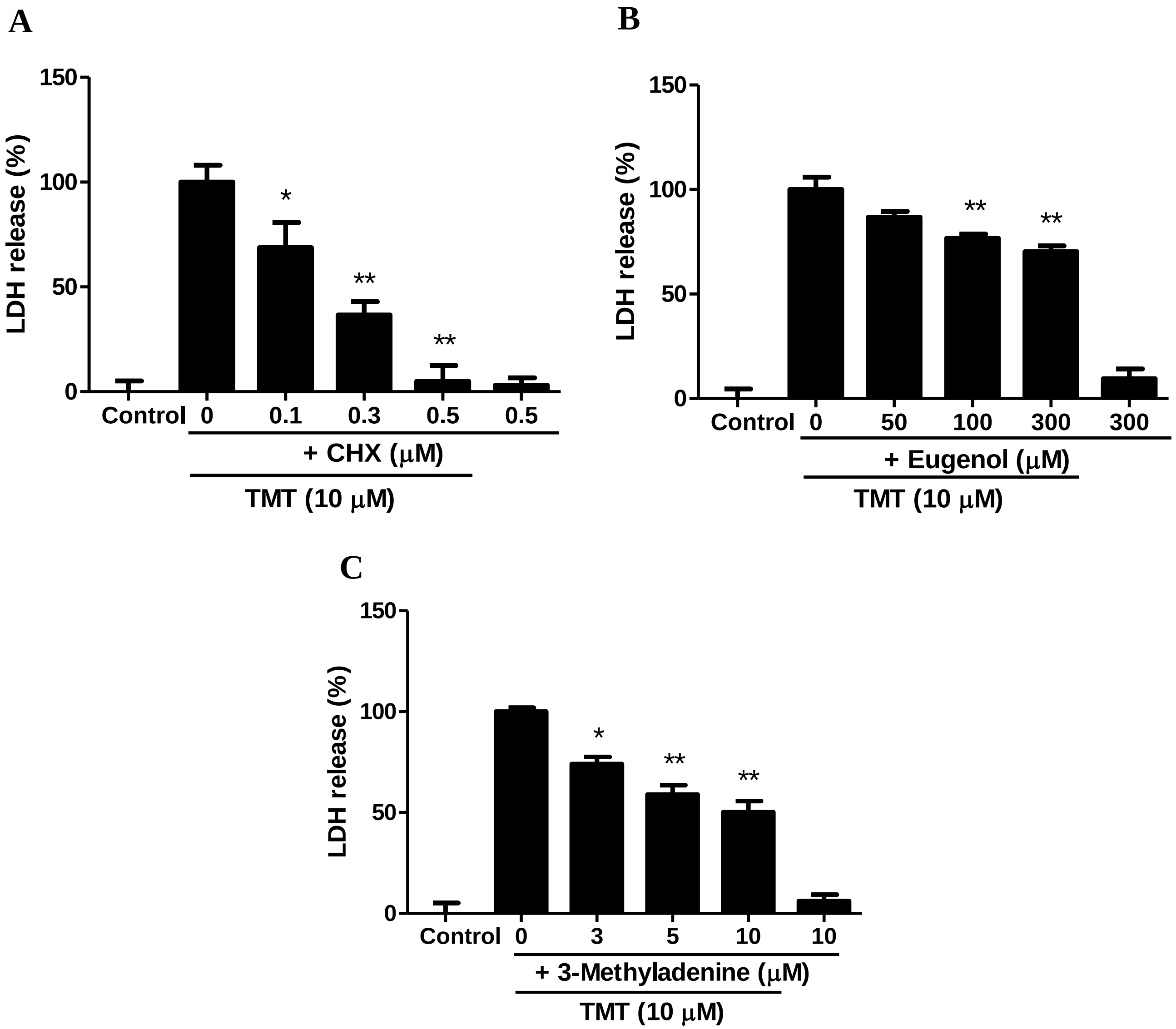

Supplemental Figure S3

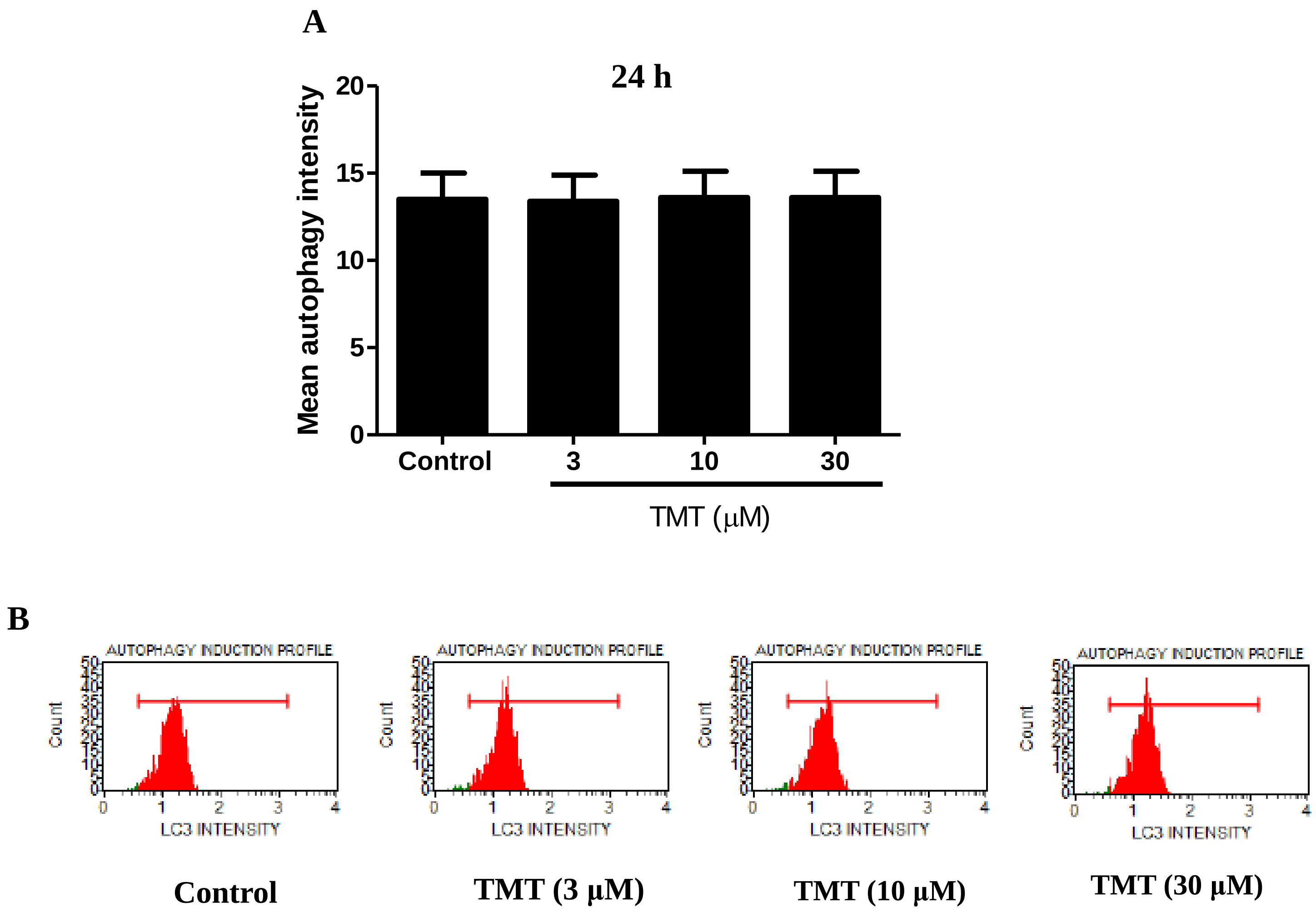

Supplemental Figure S4

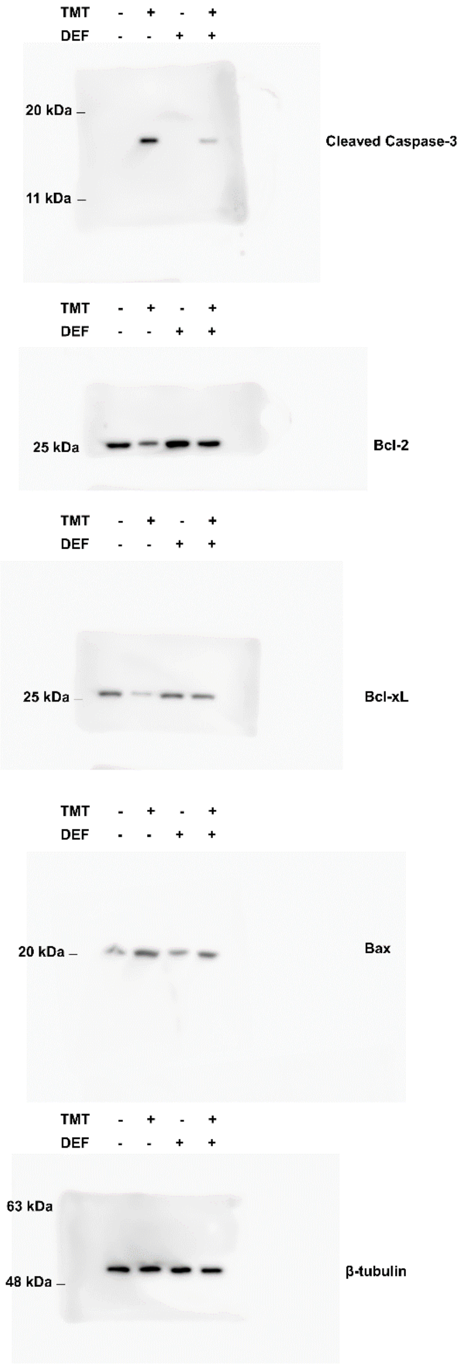

Legends of supplemental Figures

Figure S1. Excitotoxicity induced by NMDA (0.5 mM), KA (0.3 mM), and glutamate (5 mM) was not present at 24 h and 48 h after TMT treatment in SH-SY5Y cells. Cytotoxicity was evaluated using a LDH release assay. Data represents the mean ± SEM. Experiments were performed at least three times, with similar results.

Figure S2. New protein synthesis inhibitor, cycloheximide (CHX), phenylpropanoid compound of clove oil, eugenol and autophagy inhibitor, 3-methyladenine attenuate TMT-induced toxicity in SH-SY5Y cells. Data represents the mean ± SEM. *$p<0.05$ and **$p<0.01$ compared to 10- μM TMT-treated group (n=4). Experiments were performed at least three times, with similar results.

Figure S3. Effects of TMT on autophagic LC3 intensity in SH-SY5Y cells. Mean autophagy intensity did not alter at the 3, 10, 30 μM concentration of TMT at 24 h (A). Representative flow cytometry data showed in B. Data represents the mean ± SEM. Experiments were performed at least three times, with similar results.

Figure S4. Whole western blot photos of cleaved caspase-3, Bcl-2, Bcl-xL, and Bax assays induced by 10 μM TMT with or without 100 μM deferoxamine in SH-SY5Y cells. β-tubulin was used as a control. DEF : deferoxamine.
